# Supplementary material for: Characteristics of Cellulose Nanofibrils from Transgenic Trees with Reduced Expression of Cellulose Synthase Interacting 1
Source: Nanomaterials (Basel). 2022 Oct 2;12(19):3448. doi: 10.3390/nano12193448 (PMC9565832; doi:10.3390/nano12193448)
Supplement: Supplementary file 1 [file nanomaterials-12-03448-s001.zip › nanomaterials-1841392-supplementary.pdf]

Supplementary Information

Characteristics of Cellulose Nanofibrils from Transgenic Trees with Reduced Expression of Cellulose Synthase Interacting 1

Simon Jonasson [1], Anne Bünder [2], Linn Berglund [1], Totte Niittylä [2] and Kristiina Oksman [1,3,4,*]

[1] Division of Materials Science, Luleå University of Technology, 97187 Luleå, Sweden
[2] Umeå Plant Science Centre, Department of Forest Genetics and Plant Physiology, Swedish University of Agricultural Sciences, 90183 Umeå, Sweden
[3] Mechanical & Industrial Engineering, University of Toronto, Toronto, ON M5S 3G8, Canada
[4] Wallenberg Wood Science Centre (WWSC), Luleå University of Technology, 97187 Luleå, Sweden
* Correspondence: kristiina.oksman@ltu.se

Table S1. Chemical composition of wood cell wall [1].

Tree ID	Total lignin [%]	Hemicelluloses [%]	Cellulose [%]
WT	21.5 (±0.5)	21.4 (±1.0)	40.1 (±0.7)
T1	20.2 (±0.5)	19.2 (±1.1)	37.1 (±0.3)
T2	20.5 (±1.1)	20.3 (±1.5)	40.4 (±1.0)

Reference

1. Bünder, A.; Sundman, O.; Mahboubi, A.; Persson, S.; Mansfield, S.D.; Rüggeberg, M.; Niittylä, T. Cellulose synthase interacting 1 is required for wood mechanics and leaf morphology in aspen. *Plant J.* **2020**, *103*, 1858–1868. https://doi.org/10.1111/tpj.14873.
